# Biochemical Synthesis of Ag/AgCl Nanoparticles for Visible-Light-Driven Photocatalytic Removal of Colored Dyes

**DOI:** 10.3390/ma8052043

**Published:** 2015-04-23

**Authors:** Xiyun Zhao, Jie Zhang, Binsong Wang, Amir Zada, Muhammad Humayun

**Affiliations:** 1 College of Life Science, Northeast Forestry University, Harbin 150040, China; E-Mail: zhaoxiyun1972@gmail.com; 2 Key Laboratory of Chemical Engineering Process & Technology for High-Efficiency Conversion, School of Chemistry and Material Sciences, Heilongjiang University, Harbin 150080, China; 3 Department of Chemistry, Abdul Wali Khan University Mardan, KPK, Mardan 23200, Pakistan; E-Mails: amirzada@awkum.edu.pk (A.Z.); humayunchem087@gmail.com (M.H.)

**Keywords:** silver, silver chloride, plasmon resonance, photocatalysis, dye wastewater

## Abstract

Photocatalytic removal of organic pollution such as waste colored dyes was a promising technique for environment technique. However, effective photocatalysts were needed to enhance the photocatalytic efficiency. Ag/AgCl was regarded as high performance catalyst for photocatalytic degradation. Ag/AgCl nanoparticles were biochemically prepared with metabolin of living fungi which was used as reductant and characterized by X-Ray diffraction (XRD), UV-visible spectroscopy and transmission electron microscopy (TEM). The Ag/AgCl nanoparticle composites showed spherical aggregation shape with an average size of about 3–5 nm which is well inside the quantum regime. The UV-visible study showed that Ag/AgCl nanoparticles had strong visible light absorption and exhibited excellent visible-light-driven photocatalytic performance. Photocatalytic results indicated that the obtained Ag/AgCl nanoparticles were suitable for photocatalytic removal of RhB dye under visible light irradiation. The excellent photocatalytic activities could be attributed to the quantum size nanoparticles and the Plasmon resonance of Ag/AgCl composites.

## 1. Introduction

As the backbone for the economy of developed countries, dye industry plays significant role in spinning and other light industry, but brings some pollutions at the same time due to the improper discharge and other reasons [1]. The industrial effluents which contain some dyes cause much environment issues for their adverse effect on the aquatic and terrestrial life because of the water eutrophication by dyes and the presence of colored substances prevents the penetration of sunlight necessary for photosynthesis [2,3,4]. To solve this, a number of physical, chemical and biological methods have been used to remove pollutants from the industrial effluents but none of methods can be used to remove all the pollutants at the same time. Effective ways should be developed to solve this environment problem.

Color substances absorb visible radiation and can be effectively used as photosensitizers in photocatalytic reactions. And also, many photochemical reactions results in the degradation of organic compounds into CO_2_ and water in the presence of a suitable conductive substrate. The use of an effective photocatalyst and the selection of optimum working parameters result in the complete removal of color substances. Photocatalytic technique is believed to be the most effective way to remove dye pollutants in water. A number of researchers have used TiO_2_ and ZnO because their compounds have large band gaps, low cost, non-toxic and lower extensive recombination [5,6,7]. Moreover, exhaust TiO_2_ could be reused for further degradation without any decrease in its efficiency because the pore structure of these compounds remains unaffected when the adsorbed dye is desorbed [8,9]. For example Mittal *et al*. [10] studied the UV degradation of Amaranth dye in the presence of H_2_O_2_ and TiO_2_. Their experimental data revealed that the degradation efficiencies of UV, UV + H_2_O_2_, UV + TiO_2_ and (UV + TiO_2_ + H_2_O_2_) were 17%, 26%, 38% and 64%, respectively. Chung and Chen [11] investigated the photocatalytic decolourization of reactive violet 5 dye in the presence of TiO_2_ at pH 4 and the efficiency of degradation was excellent at room temperature. However, these UV-light induced photocatalysts still have such shortcomings as the use of only 5% of the total visible light. Although the visible light driven photocatalysts such as CdS, Fe_2_O_3_, and WO_3_ are also used to remove hazardous substances from drinking water [12,13]. However, the photocorrosion and the insufficient of reduction of them limited the extensive of utilization. Therefore, it is vital to develop effective visible-light photocatalytic materials to effectively improve the sunlight utilization [14]. 

The surface plasmon response of such noble metals as Au and Ag has been found to be excellent under visible light and exhibited high photocatalytic activity in a wide range of electromagnetic radiations [15]. Plasmon-induced Ag/AgX (Cl, Br, I) photocatalysts synthesized by ion-exchange, chemical precipitation and photo-assistant reduction are expensive and possess large particles. Prepared micrometer sized Ag/AgCl particles cause plasmon-induced electron-hole pairs to recombine before they reach the photocatalyst surface. The high recombination rate of charge carrier results in the decreased efficiency of a plasmonic photocatalytic system [16]. Owing to the rapid growth of bacterium, yeast, fungi and plants, biochemical synthesis is considered as a novel method for the bio-reduction of metal salts into nanomaterials [17,18,19,20]. Metal ions can easily be assimilated by micro-organisms and plants and converted into their respective metallic elements in a nano-regime size. The prepared nanoparticles can be collected using simple physical methods. Few works has been done on the preparation of nanomaterials using this method so far. However, Durán *et al*. [21,22,23] did lots of works about using biochemistry method to prepare nanomaterials. This inspires us to prepare Ag/AgCl nanoparticles by using our cultured *fungi*.

Based on the above consideration, we prepared Ag/AgCl nanoparticles aggregation composites with small and spherical size in the presence of *fungi* and used them effectively for the photocatalytic degradation of Rhodamine B. The synthesis of Ag/AgCl nanoparticles by this novel biochemical method are believed as a new and facile method to prepare nanoparticles.

## 2. Experimental Section

### 2.1. Preparation of Rose Bengal Medium

Rose Bengal medium was prepared by mixing of 5 g peptone, 10 g of glucose, 0.5 g of MgSO_4_·7H_2_O, 20 g of agar, 0.03 g of Bengal rose, 0.1 g of chloramphenicol and 1.0 g of NaH_2_PO_4_ in 1 L of pure deionized water. The prepared Bengal medium was boiled for 1 min with constant stirring and then sterilized by autoclaving for 20 min.

### 2.2. Preparation of Potato Dextrose Agar

The Potato Dextrose Agar (PDA) medium was prepared by thoroughly washing 200 g of sliced potato with distilled water and then boiling it in 1 L of deionized water for 30 min. The prepared soup was filtered with cheesecloth and transferred into a volumetric flask with a volume of 1 L. Deionized water was added into the flask up to make a total volume of 1 L of suspension. 20 g each of sucrose and agar powder were then added into the soup solution. The prepared medium was then sterilized by autoclaving for 20 min.

The culture of fungi was grown in the soil taken from Mountain Maoer in Heilongjiang Province.

### 2.3. Screening of Fungal Strains and Identification of Fungi

10 g of soil was mixed with 90 mL of deionized water for preparation of fungal suspension. 1 mL of this suspension was taken and spread over the Rose Bengal medium. The fungal strain was obtained by incubating the rose Bengal medium doped with bacteria for 5 days at a constant temperature of 28 °C. The obtained strains were thoroughly purified with distilled water and then vaccinated to PDA medium with 0.5 mM of AgNO_3_ solution and incubated for another 5 days at the same temperature of 28 °C. Finally, the fungal strains which could live in AgNO_3_ solution were obtained and used to prepare Ag/AgCl nanoparticles. NaCl was used as an obligatory material in the nutrient solution of fungi because it provided Cl^−^ for the synthesis of AgCl.

The totals DNA of fungi were extracted using Fungal DNA Kit supplied by Omega Bio-Tek Company in China. The collected DNA was used as the template for Polymerase Chain Reaction (PCR) expansion. The primer used for PCR expansion of ITS1:5’-TCCGTAGGTGAACCTGCGG-3’:ITS4:5’-TCCTCCGCTTATTGATATGC-3’ was supplied by Shangon Biotech (Shanghai, China). The purified PCR product was sent to Huada Gene Company (Shenzhen, China) for Gene sequencing and the results were submitted to Genbank database. Blast searching software was used for the most similar Gene sequence through comparison. Software MEGA5.2 [24] was used to through phylogenetic analysis to construct the phylogenetic tree; HZQ-F160 orbital shaking incubator was supplied by Shanghai Yiheng Science and Technology Ltd. (Shanghai, China) and Gene Amp 9700 PCR master cycler was supplied by American Applied Bio-systems (Foster City, CA, USA).

### 2.4. Preparation of Ag/AgCl Nanoparticles

Freshly prepared active fungal strains were vaccinated to 300 mL of PDA medium with 7.5 mL of 2 mM NaCl solutions with a vaccination needle. The culture of fungi exposed to AgNO_3_ and NaCl solutions was incubated for 5 days at 28 °C on an orbital shaking incubator at 120 rpm. The zymotic fluid was filtered with the help of filter paper. 200 mL of this fluid was mixed with 2 mL of 0.1 M AgNO_3_ solution and then incubated for 48 h at 28 °C on an orbital shaking incubator at 120 rpm. Finally, the reaction solution was centrifuged at 14,000 rpm for 5 min to obtain Ag/AgCl nanoparticles. The prepared Ag/AgCl nanoparticles were washed four times with distilled water to remove the impurities absorbed on the surface of Ag/AgCl nanoparticles.

### 2.5. Characterization

The isolated Ag/AgCl nanoparticles were characterized by UV-Vis spectroscopy scanning (TU-1901, Beijing Puxi Inc., Beijing, China). The spectra were recorded in the wavelength range of 200 to 800 nm with an interval of 1 nm. The ultrathin sections of fungi were cut on LEICA ULTRACUT UCT instrument (Solms, Germany) and the TEM analysis of ultrathin sections was made on TECNAIG2 instrument (The Netherlands) operated at a voltage of 80 kV. The crystallographic structure of Ag/AgCl nanoparticles was investigated with X-ray diffractometer (Bruker D8, Karlsruhe, Germany) operated at a voltage of 40 kV in a scan range of 20°–80°.

### 2.6. Photocatalytic Test

The photocatalytic activity of the prepared Ag/AgCl nanoparticles for the photodegradation of RhB in an aqueous solution was evaluated by in a home-built reactor at ambient temperature (25 °C) under visible light irradiation. A 150 W Xe lamp with an IR filter was used as a solar light source. The visible light obtained by an optical filter (λ > 420 nm) was used to filter the UV light. The focused intensity on the flask was both ca. 10 mW/cm^−2^. The reactor was open to the ambient air in order to reach the air-equilibrated condition. The concentration of RhB is 5 mg/L. For each run, 0.1 g of photocatalyst powders was dispersed in 20 mL of 5 mg/L RhB aqueous solution. Prior to the beginning of irradiation, the mixture was allowed to equilibrate in the dark with stirring for 30 min. Absorbance of irradiated samples was determined immediately after irradiation and removal of the powders by centrifugation and filtration. The photocatalytic performance of the obtained composite was also compared with N-TiO_2_ sample. The N-TiO_2_ was prepared by calcined commercial anatase TiO_2_ particles (Aladdin) in NH_3_ at 400 °C for 2 h. The photodegradation of RhB was determined by UV-vis absorbance at λ_max_ = 552 nm.

## 3. Results and Discussion

### 3.1. Identification of Fungi

**Figure 1:**
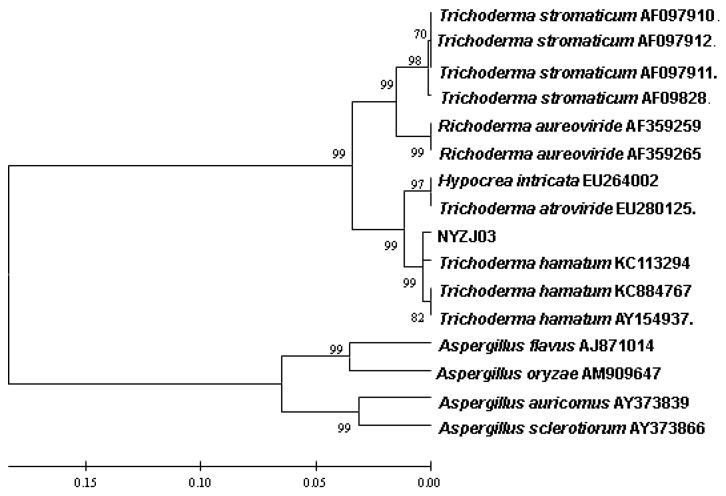
Phylogenetic tree of obtained fungal strains.

The as-prepared *fungi* used for our present work were separated and cultured by ourselves in ways which were not used by any other researcher so far. In order to identify the fungi of us, the cultured pure fungal strains were accredited against the commonly used standard [25]. The fungal strains were PCR expanded by using the DNA of fungal strain genome as the template. The ITS serial number of our fungal strains was 558 bp in contrast to all the serial numbers in GenBank as determined using BLAST program to find the nucleotide homology and the similar base sequence. The ITS number of known strain with high homology was chosen and the ITS number of our strain’s was used to construct the phylogenetic tree with the help of software MEGA5.2. 1000 times bootstrap was done to inspect the reliability of the phylogenetic tree. The strains of NYZJ03 formed the same ethnic groups with *Trichoderma hamatum* and the homology went up to 99.5% as shown in Figure 1. Our obtained fungus was considered to be *Trichoderma hamatum* from the result of morphological characteristics, homology of serials and the phylogenetic tree of fungal strains

### 3.2. XRD Analysis

**Figure 2:**
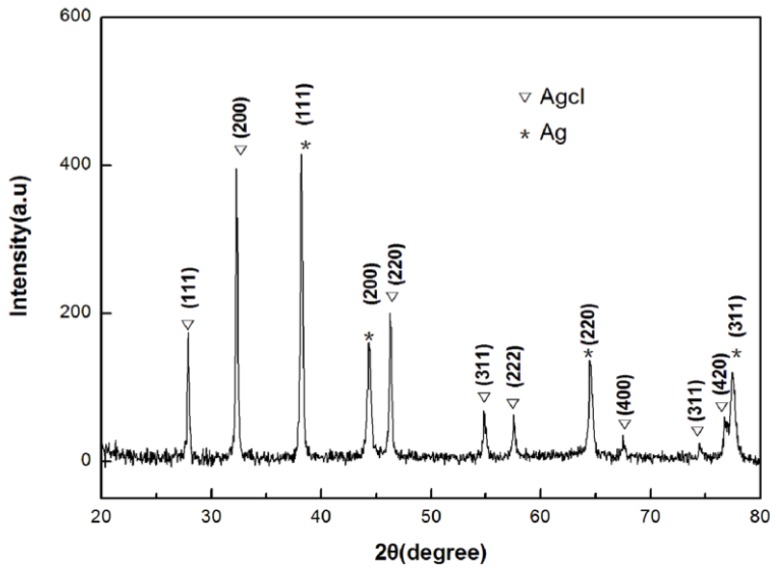
X-ray diffraction (XRD) pattern of Ag/AgCl nanoparticles.

Ag/AgCl nanoparticles were characterized using X-ray diffractometer (Bruker D8, Karlsruhe, Germany) with Cu-Kα radiation operated at a voltage of 40 kV in the scan range of 20 to 80 degree. As shown in Figure 2. The characteristic XRD peaks at 38.2°, 44.6° and 75.2° were attributed to (111), (200), and (311) reflection planes for cubic Ag (JCPDS No. 65-2871). The clear and dominant eight peaks at 27.8°, 32.2°, 46.2°, 54.9°, 57.6°, 67.4°, 74.5° and 76.7° were attributed to planes (111), (200), (220), (311), (222), (400), (331), and (420) of cubic phase of AgCl crystal (JCPDS No. 31-1238). No other characteristic peaks could be attributed to impurities which indicated the high purity of Ag/AgCl. The strong and narrow diffraction peaks reveal the highly crystalline [26]. The particle size of Ag and AgCl is calculated by Scherrer equation d = Kλ/βcosθ, which d is the mean size of the ordered (crystalline) domains; K is a dimensionless shape factor, with a value close to unity. The shape factor has a typical value of about 0.9, but varies with the actual shape of the crystallite; λ is the X-ray wavelength; β is the line broadening at half the maximum intensity (FWHM), after subtracting the instrumental line broadening, in radians. This quantity is also sometimes denoted as Δ(2θ); θ is the Bragg angle of the main crystal plane. We chose (111) of Ag and (200) of AgCl to calculate the particle size. The calculated particle size is 5.27 and 4.53 nm for Ag and AgCl, respectively.

### 3.3. TEM and HRTEM of Ag/AgCl Nanoparticles

**Figure 3:**
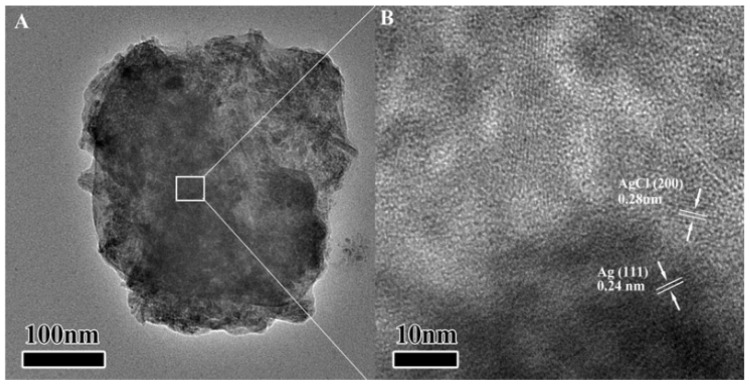
(**A**) Transmission electron microscopy (TEM) and (**B**) high-resolution transmission electron microscopy (HRTEM) images of Ag/AgCl nanoparticles.

**Figure 4:**
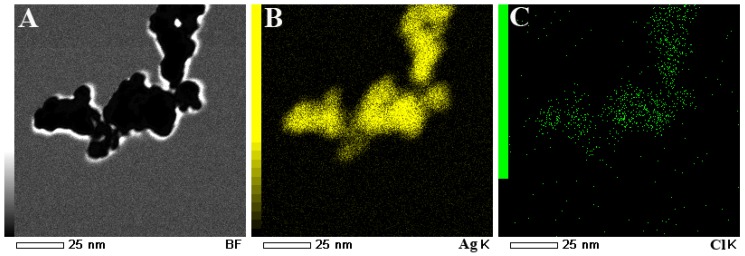
TEM-Energy Dispersive Spectroscopy (EDS) mapping of Ag/AgCl composites. (**A**) TEM image; (**B**) Mapping of Ag; (**C**) Mapping of Cl.

The living fungi could produce some substances which have the property to reduce Ag^+^ to Ag^0^ nanoparticles. Durán *et al*. [22,23] has been proven that the free *cysteine* produced by *laccase* could be as a reducing group to reduce Ag^+^. Thus, in this work, we believe that the reductant of Ag nanoparticles formation are *cysteine* and other reductive amino acids. Moreover, NaCl and chloramphenicol in the form of electrolytes are used as source of chlorine for the preparation of AgCl. Fresh AgCl and Ag nanoparticles were mixed to form Ag/AgCl composites. The Ag/AgCl composites were regarded as very good plasmon response materials for photocatalysis. The external morphology of Ag/AgCl nanoparticles was studied with TECNAIG2 instrument (Philips, Eindhoven, The Netherlands) at 80 kV. TEM and HRTEM images of Ag/AgCl composites were shown in Figure 3. TEM image in Figure 3A shows that the nanoparticles are almost spherical with approximately 3–5 nm in size and these nanoparticles aggregate with each other. This is consisting with the result by Scherrer equation from XRD peaks (5.27 and 4.53 nm for Ag and AgCl, respectively). HRTEM image in Figure 3B further investigated the Ag/AgCl composite material. The lattice fringes corresponding to (111) (d111 = 0.24 nm) were the clear crystallographic planes of Ag. The lattice fringes corresponding to (200) (d200 = 0.28 nm) were attributed to crystallographic planes of AgCl. The closed and compact interface interaction of Ag and AgCl could also be observed from HRTEM image. TEM-EDS further indicated the formation of Ag/AgCl junction composite, as shown in Figure 4, that Cl dispersed uniformly in the composite. The content of AgCl in the composite is about 12 mol% from EDS. This result clearly shows that the Ag/AgCl heterogeneous junctions are formed in the composite material which favor the charge transport and separation and improve the photocatalytic activity of Ag/AgCl nanomaterials [27].

### 3.4. UV–vis. Diffuse Reflectance Spectra

**Figure 5:**
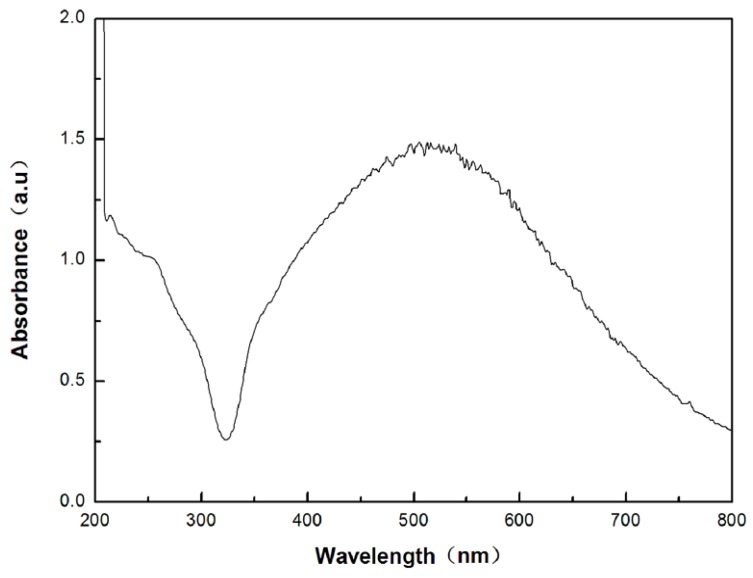
UV-visible absorption spectra of Ag/AgCl nanoparticles.

It could be seen from Figure 5, there were strong absorption in both ultraviolet and visible-light regions of electromagnetic radiations. The absorption band between 200 and 350 nm could be attributed to the characteristic absorption of AgCl composite which possesses a direct band gap of 5.6 eV and an indirect band gap of 3.25 eV. The strong absorption band in the visible-light region was attributed to the surface plasmon resonance of Ag nanoparticles. Due to the small size of silver nanoparticles in the quantum regime, the sample exhibited a stronger absorption at 400–700 nm. The strong absorption of visible light was responsible for visible-light-driven photocatalytic activity of Ag/AgCl.

### 3.5. Photocatalytic Activity

**Figure 6:**
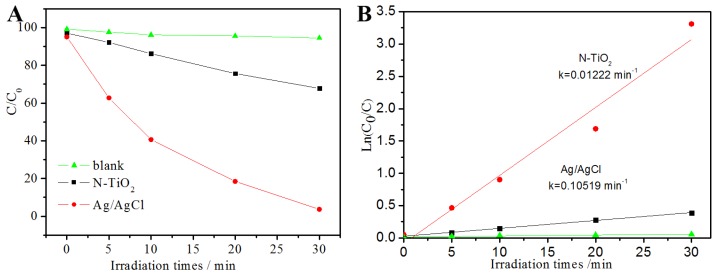
(**A**) Photodecomposition and rate of photodecomposition of RhB dyes over Ag/AgCl composites, N-TiO_2_ and (**B**) the blank experiment without photocatalysts.

Rhodamine B (RhB) was a carcinogenic chemical dye and it was often used as a tracer to determine the rate and direction of water flow [28,29,30]. RhB was used as a common modal to evaluate the performance of photocatalysts. The photocatalytic activities of Ag/AgCl nanoparticles for degradation of RhB were shown in Figure 6, in which C_0_ is the equilibrium concentration of RhB at the equilibrium adsorption state and C was the concentration of RhB after visible light irradiation. Prior to visible-light irradiation, RhB and Ag/AgCl solutions were kept in the dark for 30 min to obtain the equilibrium adsorption state. When RhB solution was exposed to the visible light for 30 min, there was no apparent change could be observed in the concentration of RhB. The concentration of the RhB solution was slightly decreased. However, when Ag/AgCl nanoparticles was mixed and then exposed to visible light under the same conditions, excellent photocatlaytic performance was investigated 96% of RhB dye was decomposed, which was much higher than the reported work [31] and that of N-TiO_2_ (in comparison to what is reported in reference [8]). The rate constant of N-TiO_2_ and Ag/AgCl shown in Figure 6B is −0.01222 and −0.10519 min^−^^1^, respectively. The excellent photocatalytic performance was mainly attributed to the closed junctions and the synergistic plasmon response of Ag and AgCl.

## 4. Conclusions

Summarily, it could be seen from the presentation above that Ag/AgCl nanoparticle composites could be biochemically prepared with the help of living fungi. The obtained Ag/AgCl nanoparticle composites showed excellent morphology and spherical shape with an average size of about 3–5 nm. The UV-visible study showed that Ag/AgCl nanoparticles have strong visible light absorption and exhibit excellent visible-light-driven photocatalytic performance. This property was utilized in the degradation of carcinogenic of RhB that after 30 f exposure to sun light the degradation rate was almost 96% indicating the good property. This biochemical method of synthesizing nanomaterials opens a new gate for the strategies and applications of nanoparticle composite materials. 

## References

[1] Rafatullah M., Sulaiman O., Hashim R., Ahmad A. (2010). Adsorption of methylene blue on low-cost adsorbents: A review. J. Hazard. Mater..

[2] Forgacs E., Cserhati T., Oros G. (2004). Removal of synthetic dyes from wastewaters: A review. Environ. Int..

[3] Robinson T., McMullan G., Marchant R., Nigam P. (2001). Remediation of dyes in textile effluent: A critical review on current treatment technologies with a proposed alternative. Bioresour. Technol..

[4] Alinsafi A., Evenou F., Abdulkarim E.M., Pons M.N., Zahraa O., Benhammou A., Yaacoubi A., Nejmeddine A. (2007). Treatment of textile industry wastewater by supported photocatalysis. Dyes Pigments.

[5] Qu Y., Zhou W., Pan K., Tian C., Ren Z., Dong Y., Fu H.G. (2010). Hierarchical anatase TiO_2_ porous nanopillars with high crystallinity and controlled length: An effective candidate for dye-sensitized solar-cells. Phys. Chem. Chem. Phys..

[6] Tian C., Li W., Zhang Q., Pan K., Fu H.G. (2011). Controllable fabrication of various ZnO micro/nanostructures from a wire-like Zn–EG–AC precursor via a facile solution-based route. Mater. Res. Bull..

[7] Tian G., Chen Y., Zhou W., Pan K., Dong Y., Tian C., Fu H. (2011). Facile solvothermal synthesis of hierarchical flower-like Bi_2_MoO_6_ hollow spheres as high performance visible-light driven photocatalysts. J. Mater. Chem..

[8] Chen X., Mao S.S. (2007). Semiconductor-based photocatalytic hydrogen generation. Chem. Rev..

[9] Asahi R., Morikawa T., Ohwaki T., Aoki K., Taga Y. (2001). Visible-light photocatalysis in nitrogen-doped titanium oxides. Science.

[10] Gupta V.K., Jain R., Mittal A., Saleh T.A., Nayak A., Agawal S., Sikarwar S. (2012). Photo-catalytic degradation of toxic dye amaranth on TiO_2_/UV in aqueous suspensions. Mater. Sci. Eng. C.

[11] Chung Y.C., Chen C.Y. (2009). Degradation of azo dye reactive violet 5 by TiO_2_ photocatalysis. Envir. Chem. Lett..

[12] Hwang D.W., Kim J., Park T.J., Lee J.S. (2002). Mg-doped WO_3_ as a novel photocatalyst for visible light-induced water splitting. Catal. Lett..

[13] Hu C., Hu X.X., Wang L.S., Qu J.H., Wang A.M. (2006). Visible-light-induced photocatalytic degradation of azodyes in aqueous AgI/TiO_2_ Dispersion. Environ. Sci. Technol..

[14] Chen X., Liu L., Yu P.Y., Mao S.S. (2011). Increasing solar absorption for photocatalysis with black hydrogenated titanium dioxide nanocrystals. Science.

[15] Xin B., Jing L., Ren Z., Wang B., Fu H. (2005). Effects of simultaneously doped and deposited Ag on the photocatalytic activity and surface states of TiO_2_. J. Phys. Chem. B.

[16] Bi Y., Ye J. (2009). *In situ* oxidation synthesis of Ag/AgCl core–shell nanowires and their photocatalytic properties. Chem. Commun..

[17] Ahmad A., Mukherjee P., Senapati S., Mandal M., Khan M.I., Kumar R., Sastry M. (2003). Extracellular biosynthesis of silver nanoparticles using the fungus Fusarium oxysporum. Colloids Surf. B Biointer..

[18] Jain N., Bhargava A., Majumdar S., Tarafdar J.C., Panwar J. (2011). Extracellular biosynthesis and characterization of silver nanoparticles using Aspergillus flavus NJP08: A mechanism perspective. Nanoscale.

[19] Acevedo F., Pizzul L., Castillo M.P., González M.E., Cea M., Gianfreda L., Diez M.C. (2012). Degradation of polycyclic aromatic hydrocarbons by free and nanoclay-immobilized manganese peroxidase from Anthracophyllum discolor. Chemosphere.

[20] Liu X.L., Zhu P.X., Gao Y.F., Jin R.H. (2012). Polyamine-promoted growth of one-dimensional nanostructure-based silica and its feature in catalyst design. Materials.

[21] Durán N., Marcato P.D., Souza G., Alves O.L., Esposito E. (2007). Antibacterial effect of silver nanoparticles produced by fungal process on textile fabrics and their effluent treatment. J. Biomed. Nanotechnol..

[22] Durán N., Marcato P.D., Durán M., Yadav A., Gade A., Rai M. (2011). Mechanistic aspects in the biogenic synthesis of extracellular metal nanoparticles by peptides, bacteria, fungi, and plants. Appl. Microbiol. Biotechnol..

[23] Durán N., Cuevas R., Cordi L., Rubilar O., Diez M.C. (2014). Biogenic silver nanoparticles associated with silver chloride nanoparticles (Ag@AgCl) produced by laccase from Trametes versicolor. Springer Plus.

[24] Tamura K., Peterson D., Peterson N., Stecher G., Nei M., Kumar S. (2011). MEGA5: molecular evolutionary genetics analysis using maximum likelihood, evolutionary distance, and maximum parsimony methods. Mol. Biol. Evol..

[25] Cheung Y.C., Liu X.X., Wang W.Q., Wu J.Y. (2015). Ultrasonic disruption of fungal mycelia for efficient recovery of polysaccharide–protein complexes from viscous fermentation broth of a medicinal fungus. Ultrason Sonochem..

[26] Wang P., Huang B., Lou Z., Zhang X., Qin X., Dai Y., Zheng Z., Wang X. (2010). Synthesis of highly efficient Ag@AgCl plasmonic photocatalysts with various structures. Chem. Eur. J..

[27] Wang P., Huang B., Qin X., Zhang X., Dai Y., Wei J., Whangbo M.H. (2008). Ag@AgCl: A highly efficient and stable photocatalyst active under visible light. Angew. Chem. Int. Ed..

[28] Ferrari C., Chen H., Lavezza R., Santinelli C., Longo I., Bramanti E. (2013). Photodegradation of Rhodamine B Using the Microwave/UV/H_2_O_2_: Effect of Temperature. Int. J. Photoenergy.

[29] Yang S., Huang Y., Wang Y., Yang Y., Xu M., Wang G. (2012). Photocatalytic degradation of Rhodamine B with H_3_PW_12_O_4_0/SiO_2_ sensitized by H_2_O_2_. Int. J. Photoenergy.

[30] Luan J., Xu Y. (2013). Photophysical property and photocatalytic activity of new Gd_2_InSbO_7_ and Gd_2_FeSbO_7_ compounds under visible light irradiation. Int. J. Mol. Sci..

[31] Xu H., Li H., Xia J., Yin S., Luo Z., Liu L., Xu L. (2011). One-pot synthesis of visible-light-driven plasmonic photocatalyst Ag/AgCl in ionic liquid. ACS Appl. Mater. Int..

